# Deciphering Ligand Specificity of a *Clostridium thermocellum* Family 35 Carbohydrate Binding Module (*Ct*CBM35) for Gluco- and Galacto- Substituted Mannans and Its Calcium Induced Stability

**DOI:** 10.1371/journal.pone.0080415

**Published:** 2013-12-06

**Authors:** Arabinda Ghosh, Ana Sofia Luís, Joana L. A. Brás, Neeta Pathaw, Nikhil K. Chrungoo, Carlos M. G. A. Fontes, Arun Goyal

**Affiliations:** 1 Department of Biotechnology, Indian Institute of Technology Guwahati, Guwahati, Assam, India; 2 CIISA-Faculdade de Medicina Veterinaria, Avenida da Universidade Técnica, Lisbon, Portugal; 3 North Eastern Hill University, Shillong, Meghalaya, India; George Washington University, United States of America

## Abstract

This study investigated the role of CBM35 from *Clostridium thermocellum* (*Ct*CBM35) in polysaccharide recognition. *Ct*CBM35 was cloned into pET28a (+) vector with an engineered His_6_ tag and expressed in *Escherichia coli* BL21 (DE3) cells. A homogenous 15 kDa protein was purified by immobilized metal ion chromatography (IMAC). Ligand binding analysis of *Ct*CBM35 was carried out by affinity electrophoresis using various soluble ligands. *Ct*CBM35 showed a manno-configured ligand specific binding displaying significant association with konjac glucomannan (*K*_a_ = 14.3×10^4^ M^−1^), carob galactomannan (*K*_a_ = 12.4×10^4^ M^−1^) and negligible association (*K*_a_ = 12 µM^−1^) with insoluble mannan. Binding of *Ct*CBM35 with polysaccharides which was calcium dependent exhibited two fold higher association in presence of 10 mM Ca^2+^ ion with konjac glucomannan (*K*_a_ = 41×10^4^ M^−1^) and carob galactomannan (*K*_a_ = 30×10^4^ M^−1^). The polysaccharide binding was further investigated by fluorescence spectrophotometric studies. On binding with carob galactomannan and konjac glucomannan the conformation of *Ct*CBM35 changed significantly with regular 21 nm peak shifts towards lower quantum yield. The degree of association (*K*_a_) with konjac glucomannan and carob galactomannan, 14.3×10^4^ M^−1^ and 11.4×10^4^ M^−1^, respectively, corroborated the findings from affinity electrophoresis. The association of *Ct*CBM35with konjac glucomannan led to higher free energy of binding (ΔG) −25 kJ mole^−1^ as compared to carob galactomannan (ΔG) −22 kJ mole^−1^. On binding *Ct*CBM35 with konjac glucomannan and carob galactomannan the hydrodynamic radius (R_H_) as analysed by dynamic light scattering (DLS) study, increased to 8 nm and 6 nm, respectively, from 4.25 nm in absence of ligand. The presence of 10 mM Ca^2+^ ions imparted stiffer orientation of *Ct*CBM35 particles with increased R_H_ of 4.52 nm. Due to such stiffer orientation *Ct*CBM35 became more thermostable and its melting temperature was shifted to 70°C from initial 50°C.

## Introduction

Hydrolytic enzymes and their enhanced polysaccharide specificity often improve by appended non catalytic carbohydrate binding module either at their N or C terminal ends. A major portion of plant carbohydrate reservoir is composed of hemicelluloses such as the polymer and oligomer of xylose, mannose, arabinose etc. apart from celluloses. Polysaccharide recognition, binding and enhanced catalysis of hydrolytic enzymes truly facilitates by non catalytic modular carbohydrate binding modules. Carbohydrate binding modules (CBMs) are classified into 67 distinguished families based on sequence similarity (http://www.cazy.org/Carbohydrate-Binding-Module). CBMs are found in the protein both as appended with hydrolytic enzymes such as cellulase, mannanase, xylanase, as non hydrolytic functions, as scaffoldings and as peptide with non catalytic functions with variety of hydrolytic enzyme complex (cellulosome) [1]. Family 35 carbohydrate binding module is often appended to glycoside hydrolase family 26 (GH26) and GH5 mannanases [2]–[4], xylanases (GH30) [5] which significantly alter the polysaccharide specificity for plant cell wall polysaccharides such as galactomannan, glucomannan, mannan and glucouronoxylan. Three dimensional structures of CBM35 family generally have the dominance of β-sheet secondary structures with a jelly roll topology [5]. CBM35 usually accommodates the polysaccharides utilizing a planer surface of aromatic side chains which interact with the flat chains of manno-configured carbohydrate residues. This form of conformation is known as type B module [5]. Polysaccharide binding significantly alters the conformation of CBM35 by changing the loop orientation containing amino acid residues which facilitate to create a suitable binding space for polysaccharide accommodation [6]. Since the binding specificity depends on the polysaccharide complexity and side chain interactions of monosaccharide a variety of diverted binding may be observed. Thus the polysaccharide specificity varies due to chain substitution and orientation of monosaccharides [7]. In earlier study, it was attributed to role of divalent cations in alteration of the domain conformation of CBMs that enhance the higher polysaccharide specificity and thermostability [8]. Thermostability one of the major concern of most of the enzyme stability and activity during industrial process and thus this binding module may restore the potentialities of the hydrolytic enzymes. Moreover, carbohydrate binding modules are used in various analytical processes. Thermostable CBMs were explored to separate cello- and xylo-oligosaccharides based on their affinity towards these carbohydrates [9]. CBM microarray technique is more pronounced that replaced the conventional DNA microarray. This method is simple, effective and an alternative to various conventional microarray technologies [10]. Moreover, substantial rise in effective enzyme catalysis process may induce by this carbohydrate binding module will meet the requirement for carbohydrate fermentation to most demanding biofuel [11].

Our work is focused on the functional and conformational properties of CBM35 from *Clostridium thermocellum* ATCC 27405 (*Ct*CBM35) upon polysaccharide and cation binding. *Ct*CBM35 usually display the specificity towards manno-configured polysaccharides. Mannose has single stereochemical difference from glucose (at 2′-OH site) in the manno-configured polysaccharides which made it less rigid structural conformation. *Ct*BM35 has varying degree of affinity for lesser and higher galactose and glucose substituted polysaccharides. The affinity electrophoresis, fluorescence measurements and dynamic light scattering were employed to analyze both qualitative and quantitative binding of *Ct*BM35 with manno-configured polysaccharides. The structural variations of *Ct*BM35 due to Ca^2+^ ion binding and the alterations of domain conformations were described in this work.

## Materials and Methods

### Gene amplification and cloning of *Ct*CBM35

The open reading frame (ORF) of *Ct*CBM35 was amplified from the genomic DNA of *Clostridium thermocellum* ATCC 27405 using a forward primer containing *Nhe*I restriction site: CAC**GCTAGC**GCATATTCCCTTCCTG and a reverse primer with *Xho*I restriction site: CAC**CTCGAG**TTAAAGTTCATCCAAGCTG. The PCR conditions were followed as Mg^2+^ ions (2.5 mM), dNTPs (2 mM), primers (1.5 µM), 1.0 µl of Taq DNA polymerase (1 µl of 1Unit µl^−1^) and 1 µl of genomic DNA of *C. thermocellum*. The PCR amplification cycles used were: denaturation at 94°C for 4 min followed by 30 cycles of denaturation at 94°C for 30 s, annealing at 55°C for 60 s and extension at 72°C for 2 min and final extension at 72°C for 10 min. The amplified products were run on 0.8% agarose gel and further purified by gel extraction (Qiagen kit.) The amplified products were then digested with *Nhe*I-*Xho*I restriction enzymes and cloned into *Nhe*I/*Xho*I restricted pET-28a (+) expression vectors containing kanamycin as resistant marker respectively which resulted in cloned plasmids (pCBM35). Thereafter, *E. coli* DH5α cells were transformed with above cloned plasmids. The transformed cells were grown on LB [12] agar plates supplemented with kanamycin (50 µg ml^−1^) at 37°C for growth of recombinant clones. Positive clones were selected by restriction digestion analysis of the resulting cloned plasmids.

### Protein expression and purification

*E. coli* BL-21(DE3) (Novagen) cells were used for expressing *Ct*CBM35 as described elsewhere [12]. *E. coli* BL-21 (DE3) cells were transformed with pCBM35. The cells were grown similarly as described in Section 2.1, with incubation at 37°C and shaking at 180 rpm till mid-exponential phase (A600_nm_≈0.6). The cells were induced with 1.0 mM isopropyl-1-thio-β-D-galactopyranoside (IPTG) for hyper-expression of recombinant protein at optimized expression conditions of 24°C at 180 rpm for 16 h. The cells were harvested at 9,000 *g* and the resulting pellet was resuspended in Sodium phosphate buffer (50 mM, pH 7.0). Then the cells were sonicated (Vibra cell, Sonics) on ice for 16 min (9 s on/off pulse) with further centrifugation (19,000 g, 30 min, 4°C) to get the crude cell free protein in the supernatant. Hybrid protein containing *Ct*CBM35 appended by His_6_ tag were purified in a single step by immobilized metal ion affinity chromatography (IMAC) using Ni-Sepharose columns (HiTrap Chelating, GE Healthcare) as recommended by the manufacturer. The purity and molecular mass of recombinant proteins were verified by SDS-PAGE [13].

### Binding assay of family 35 Carbohydrate Binding Module (*Ct*CBM35)

The polysaccharide binding capability of the non-catalytic modules was determined by visualizing the adsorption to soluble polysaccharides using gel retardation in native polyacrylamide gel electrophoresis containing the polysaccharides as described earlier by Takeo, (1990) [14]. Purified *Ct*CBM35 (18 µg) was assayed with soluble polysaccharides such as carob galactomannan, konjac glucomannan, carboxymethyl cellulose, rye arabinoxylan, birchwood xylan, oatspelt xylan and glucouronoxylan. The polysaccharide samples were prepared subsequently by diluting in filtered water from (0.5%, w v^−1^) polysaccharide stock. Native polyacrylamide gels (7.5%) were prepared containing varying polysaccharide concentrations ranging from 0.0 to 1.5 (%, w v^−1^). Bovine serum albumin (BSA) sample (1.0 mg ml^−1^) was run in native gel for non-specific binding interaction. Binding study of *Ct*CBM35 with carob galactomannan and konjac glucomannan was carried out in absence and presence of Ca^2+^ ions. 10 mM Ca^2+^ ion was incorporated in 7.5% native polyacrylamide gel before *Ct*CBM35 electrophoresis. In absence of Ca^2+^ ions 7.5% native gels were prepared as control and the relative mobilities of *Ct*CBM35 were calculated.

### Binding analysis of *Ct*CBM35 with insoluble polysaccharides

The quantitative and qualitative assessment of *Ct*CBM35 binding was carried out with insoluble mannan, avicel and wheat arabinoxylan. Thirty microgram of *Ct*CBM35 in 50 mM sodium phosphate buffer, pH 7.0, was mixed with 1 mg of mannan in a final reaction volume of 200 µL. The reaction mixture was incubated for 2 h at 4°C with gentle shaking. After that the insoluble ligand was precipitated by centrifugation at 13000 *g* for 5 min. The supernatant, containing the unbound proteins, was removed and the pellet was washed three times with 200 µl of 50 mM sodium phosphate buffer pH 7.0. The bound protein from the washed pellet was eluted by boiling the polysaccharides in 200 µl of 10% (w v^−1^) SDS containing 10% (v v^−1^) β-mercaptoethanol for 10 min. The pellets of bound protein and the supernatant of unbound protein were analysed by 12% SDS-PAGE. A Bovine Serum Albumin (1 mg ml^−1^) control was set in parallel to check for any non specific binding. All the gels containing protein and no polysaccharide and the electrophoresis were performed in parallel to ensure also that no precipitation of protein occurred. For quantitative analysis the free or unbound protein concentration in un-bound fraction obtained after centrifugation was determined by Bradford method [15] and the bound protein was estimated by subtracting the free protein from the initial protein concentration. The adsorption parameters were calculated to determine the binding. If we consider [B] the bound protein concentration, [F] the unbound fraction of protein, [N] the number of binding site concentration and *K_a_* is the association constant then at equilibrium adsorption were calculated as described earlier by Gilkes *et al* (1992) [16].

### Polysaccharide binding study of *Ct*CBM35 by fluorescence spectroscopy

On binding to polysaccharides, carbohydrate binding modules undergo conformational changes and behave differently than in its unbound native form [17]. To compare the results with affinity electrophoresis, 160 µM of *Ct*CBM35 was incubated with polysaccharides *viz.*, carob galactomannan and konjac glucomannan of varying concentrations. Polysaccharide concentrations (0.01%, 0.04%, 0.06%, 0.08%, 0.15% and 0.2%, w v^−1^) from 0.5% (w v^−1^) stock solution in 100 µl reaction mixture were prepared in 50 mM sodium phosphate buffer, pH 7.0. The samples were incubated at 4°C for 2 h. The fluorescence measurements were carried out using a fluorimeter (Fluoromax 3, Horiba Scientific, USA). Emission and excitation slits were kept at 3.00 and 1.00, respectively, with 0.5 s integration time. Three scans were taken per sample along with a control to reduce the noise created by buffer and polysaccharide. All the samples were excited at λ_max_ = 295 nm with observance of emission spectra between λ_max_ = 320–400 nm. The emission spectra of all the solutions were corrected against buffer and polysaccharide solution without *Ct*CBM35 before setting the interaction study. Relative fluorescence intensities (F_o_/F, where F_o_ is initial fluorescence intensity of *Ct*CBM35 and F is final fluorescence intensity of polysaccharide *Ct*CBM35 conjugate) were plotted against polysaccharide concentration. The association constants 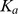
(M^−1^) of *Ct*CBM35 complex with carob galactomannan and konjac glucomannan were derived using modified Stern Volmer equation [18] as follows
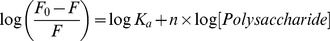



### Study of size of *Ct*CBM35 on binding with polysaccharide and Ca^2+^ by dynamic light scattering

Polysaccharide binding greatly influences the protein conformational changes. These changes may lead to more dispersion in the dynamic environment leading to higher hydrodynamic area. The binding of polysaccharide with *Ct*CBM35 was studied by dynamic light scattering (DLS). In a dynamic environment, the particles of ligand and protein molecules diffuse randomly. DLS essentially measures fluctuation in scattered light intensity due to diffusion of particles, the diffusion coefficient of the particles can be determined. The diffusion coefficient D is then related to the radius R of the particles by means of the Stokes-Einstein Equation [19]:
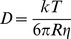

*where*, k = Boltzmann-constant, T = temperature and η = viscosity.

The hydrodynamic diameters (R_H_) of *Ct*CBM35 in presence of 0.1% (w/v) polysaccharides such as carob galactomannan and konjac glucomannan were measured by Zetasizer Nano ZS (Malvern, UK) spectrophotometer. Refractive index and viscosity of 50 mM sodium phosphate buffer were adjusted to 1.3206 and 8.945×10^−3^ g (cm s)^−1^, respectively by using SEDNTERP tool package (http://www.jphilo.mailway.com). The hydrodynamic radius of *Ct*CBM35 was also studied in presence and absence of 10 mM Ca^2+^ ions. *Ct*CBM35 was incubated at 25°C for 2 h and the extra unbound Ca^2+^ was removed by dialysis against water. The hydrodynamic radius of bound *Ct*CBM35 with Ca^2+^ conjugate was measured and compared against Ca^2+^ free *Ct*CBM35. The instrument was set to measure the absorbance at a fixed angle (θ = 90°). All the measurements were derived by deconvolution of intensity and sample autocorrelation function. Deconvolution of measured intensity was obtained using non negative least square analysis (NNLS) [19], [20] algorithm e.g. CONTIN [21], Regularization and Multiple Narrow Mode algorithms [22], [23]. These algorithms of fitting the data were included as inbuilt functions of Zetasizer Nano software package.

### Protein melting and molecular dynamics study of *Ct*CBM35 in presence of Ca^2+^ ion

Binding assays with soluble polysaccharides showed that an increase in enzyme affinity could be attributed to the presence of family 35 carbohydrate binding module. To investigate whether the presence of Ca^2+^ ion may enhance the stability of *Ct*CBM35, the stabilizing effect was studied by UV spectroscopy. Protein melting curves were generated for recombinant *Ct*CBM35 (pH 7.0, sodium phosphate buffer) by measuring in UV-absorption spectrophotometer (Varian, Cary 100) at 280 nm following the method of Dvortsov *et al.* (2009) [24]. The temperature was varied from 40–100°C using the peltier temperature controller (Cary 100-Bio, Varian) and the solutions were kept at the particular temperature for sufficient time (10 min) to attain equilibrium. *Ct*CBM35 was incubated with 10 mM CaCl_2_ at 25°C for 2 h and dialyzed against water to remove additional Ca^2+^ ion.

To investigate the role of Ca^2+^ ion in altering protein conformations a model of *Ct*CBM35 from *Clostridium thermocellum* was generated in presence of Ca^2+^ ion based on the crystal structure of closest homolog of CBM35 from *Amycolaptosis orientalis* (PDB ID: 2VZPA) by Modeller9v8 program. The homolog was identified using Blast PDB (http://www.ncbi.nlm.nih.gov/blast/Blast.cgi). The structure was energy minimized with GROMACS4.0.7 package (http://www.gromacs.org/) using steepest descent algorithm with GROMOS96 43a1 force field and simple point charge (SPC) water model [25]–[27]. Molecular dynamics (MD) simulation on the energy minimized *Ct*CBM35 model was carried out with the periodic boundary conditions applied in three dimensions to analyse the stability of the protein model. The net charge of system was neutralized by the addition of eleven sodium ions by replacing water molecules that are at least 3.50 Å from the protein surface [28]. The stable model was further visualized and analyzed in PyMOL tool.

## Results

### Cloning, expression and purification of *Ct*CBM35

**Figure 1:**
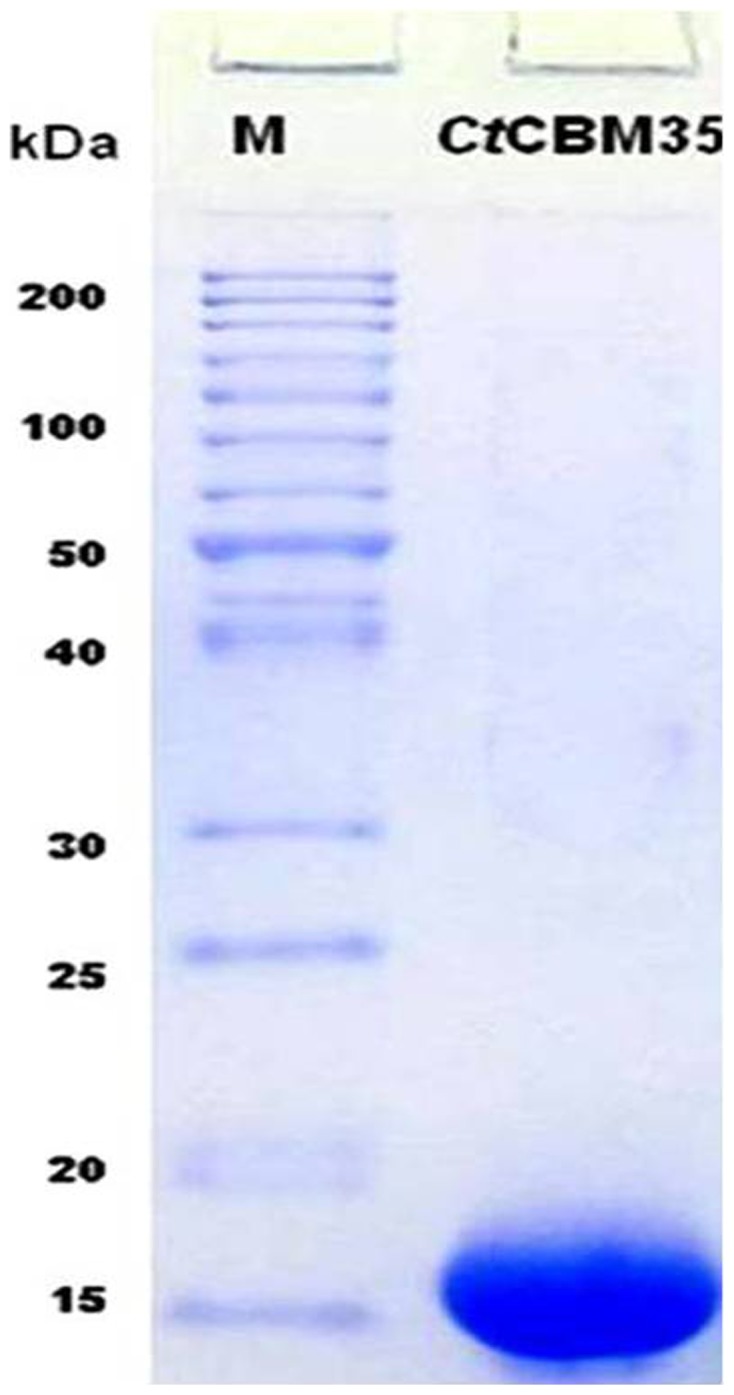
Denaturing SDS-PAGE (12%) of recombinant *Ct*CBM35 purified by IMAC.

The ORF region encoding *Ct*CBM35 was amplified by polymerase chain reaction and successfully ligated to pET28a (+) expression vector and transformed *E. coli* DH5α cells. The colonies appeared in the LB plates supplemented with 50 µg ml^−1^ kanamycin were screened for positive clones by digestion with *Nhe* I and *Xho* I restriction enzymes. Few positive clones with an insert of 420 bp and a vector fragment of 5.4 kb were obtained in 1% agarose gel electrophoresis. The transformed *E. coli* BL21 (DE3) cells by recombinant plasmids of positive clones after IPTG induction were screened for expression. The expression of *Ct*CBM35 (15 kDa protein) band was observed on 12% SDS-PAGE by comparing with long range (10–200 kDa) prestained molecular weight marker (Fermentas). The recombinant *Ct*CBM35 was purified by Ni^2+^-NTA (Immobilized metal ion chromatography) and the elution was accomplished with 300 mM Imidazole. A purified homogenous single band 15 kDa of *Ct*CBM35 appeared on 12% SDS-PAGE (Figure 1).

### Binding assay of *Ct*CBM35 with soluble polysaccharides

**Figure 2:**
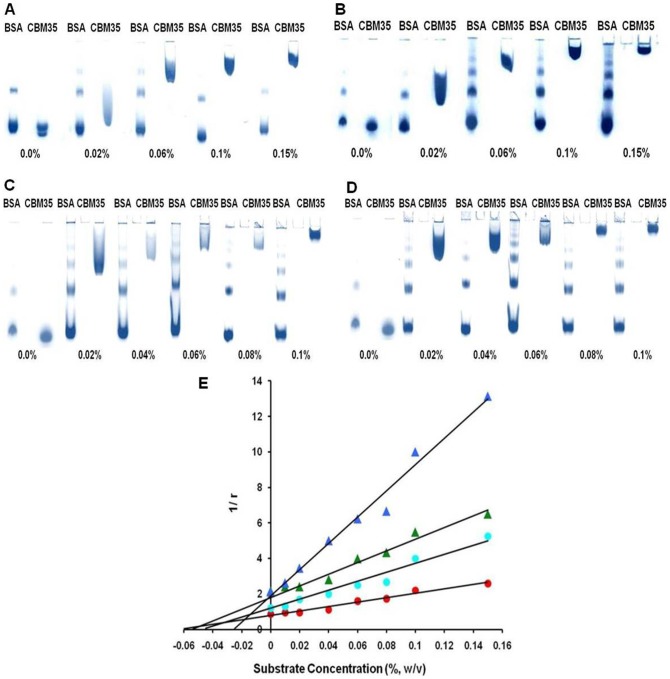
Affinity electrophoresis of *Ct*CBM35 using 7.5% native PAGE in presence of varying concentrations of (A) carob galactomannan (B) konjac glucomannan (C) 10 mM Ca^2+^ incorporated with carob galactomannan (D) 10 mM Ca^2+^ incorporated with konjac glucomannan (E) A non linear regression plot of inverse relative migration of *Ct*CBM35 (1/r) against polysaccharide concentration (%, w v^−1^), (•) carob galactomannan (in red), (▴) konjac glucomannan (in green) and (•) in presence of 10 mM Ca^2+^ ion with carob galactomannan (in light blue), (▴) in presence of 10 mM Ca^2+^ ion with konjac glucomannan (in dark blue).

To investigate the ligand binding specificity of *Ct*CBM35 the protein was expressed and purified to electrophoretic homogeneity. The affinity of *Ct*CBM35 for carob galactomannan, konjac glucomannan, rye-arabinoxylan, oat spelt xylan and lichenan was determined by affinity electrophoresis [14]. The relative mobilities of *Ct*CBM35 in presence of various soluble polysaccharides were calculated against a reference with no ligand in the affinity gel (Figure 2A & 2B). A non linear regression plot was generated between relative migration of *Ct*CBM35 against varying concentrations of ligand to calculate the association constant (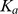) (Figure 2E). The *Ct*CBM35 displayed higher binding affinity with konjac glucomannan as compared to carob galactomannan. The association constants (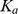) were found to be 14.3×10^4^ M^−1^ with konjac glucomannan and 12.4×10^4^ M^−1^ with carob galactomannan. No association of *Ct*CBM35 was seen with carboxymethyl cellulose, rye arabinoxylan, birchwood xylan, oatspelt xylan, glucouronoxylan (Table 1). To assess the Ca^2+^ induced affinity binding of *Ct*CBM35 with polysaccharides at their varying concentrations a similar approach was carried out by affinity electrophoresis as described earlier (Takeo et al., 1990) [14]. In presence of 10 mM Ca^2+^ ion the affinity of *Ct*CBM35 for carob galactomannan was increased to approximately 2.5 folds and the association constant (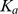) was obtained as 30×10^4^ M^−1^ and with konjac glucomannan 41×10^4^ M^−1^ (Table 1) (Figure 2C, 2D and 2E).

**Table 1:** Association constants (*K*_a_) and free energy of binding of *Ct*CBM35 from affinity electrophoresis and relative fluorescence intensities.

Polysaccharide	*K*_a_ from AE(10^4^ M^−1^)	*K*_a_ from AE (10 mM Ca^2+^)(10^4^ M^−1^)	*K*_a_ from fluorescence(10^4^ M^−1^)	Binding site (n)	Gibb's free energy (ΔG)(kJ mole^−1^)
Carob galactomannan	12.4	30	11.4	0.79	−22.0
Konjac glucomannan	14.3	41	14.3	0.80	−25.0

*AE: Affinity Electrophoresis.*

### Binding analysis of *Ct*CBM35 with insoluble polysaccharides

**Figure 3:**
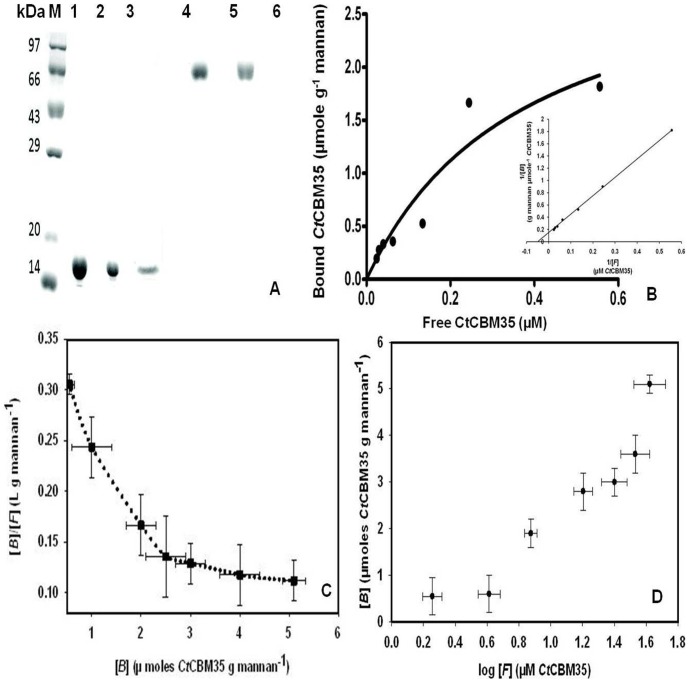
Qualitative binding of *Ct*CBM35 with insoluble mannan (A) using 12% SDS-PAGE. Lane 1: High range unstained molecular weight marker (200 kDa - 10 kDa), lane 2: Purified *Ct*CBM35, lane 3: unbound *Ct*CBM35, lane 4: bound *Ct*CBM35, lane 5: Bovine serum albumin (BSA) as control, lane 6: unbound BSA, lane 7: bound BSA. (B) Adsorption of *Ct*CBM35 to insoluble mannan. The main panel shows the equilibrium adsorption isotherm ([B] versus [F]) for *Ct*CBM35. Adsorption assay was done at 4°C, as described under methods section. Initial protein concentrations of *Ct*CBM35 were 0.2–19 µM. In the small panel showing a linear regression plot of 1/[B] versus 1/[F] concentrations to derive the association constant (*K*_a_). (C) Scatchard plot of [B]/[F] vs [B]. The curved line was fitted to data points for *Ct*CBM35 by least square regression analysis. (D) a semi-logarithmic plot ([B] vs log [F]) for adsorption data of *Ct*CBM35. In both the plots the standard errors in two dimensions are indicated by vertical and horizontal bars.

The quantitative and qualitative binding of *Ct*CBM35 with insoluble polysaccharides was assessed by adsorption isotherm analysis. *Ct*CBM35 displayed low binding with insoluble mannan as analysed by SDS-PAGE when compared with the protein in free (unbound to mannan) form and the total purified protein (Figure 3A). *Ct*CBM35 displayed no binding with avicel and wheat arabinoxylan. The saturation of insoluble mannan binding by *Ct*CBM35 was achieved approached but not to the highest protein concentration used (Figure 3B). The failure to reach saturation was detected when the same data was plotted in a semi logarithmic graph between ([B] vs log [F]). Scatchard plot of *Ct*CBM35 (Figure 3C and Figure 3D) indicated the complex binding with insoluble mannan. In this quantitative binding assessment at equilibrium the association constant (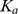) of *Ct*CBM35 with insoluble mannan was 12 µM^−1^ (Table 2). The relative equilibrium association constant *K*_r_ and the concentration of binding sites in mannan surface [N_o_] were calculated from a non linear regression plot between bound *Ct*CBM35 versus free *Ct*CBM35. The data were analyzed by GraphPad (Prism 2.0.1) software using non-linear regression analysis based on one binding site equation. The estimated values of relative equilibrium constant *K*_r_ and concentration of binding sites [N_o_] were 0.49±0.2 l g^−1^ and 0.04±0.002 µmole g^−1^, respectively (Table 2). This result suggested that *Ct*CBM35 bind to insoluble mannan less effectively as the protein binding site on the polysaccharide was less. This may be due to binding of protein to the non reducing end of the polysaccharide.

**Table 2:** Binding parameters of *Ct*CBM35 on binding with insoluble mannan derived from adsorption isotherm analysis.

Polysaccharide	*K*_r_ (l g^−1^)*	*K*_a_ (µM^−1^)	*N_o_* (µmole g^−1^)*
Mannan	0.49±0.02	12	0.04±0.002

* *values are mean ± SD (n = 3).*

### Polysaccharide binding study of *Ct*CBM35 by fluorescence spectroscopy

**Figure 4:**
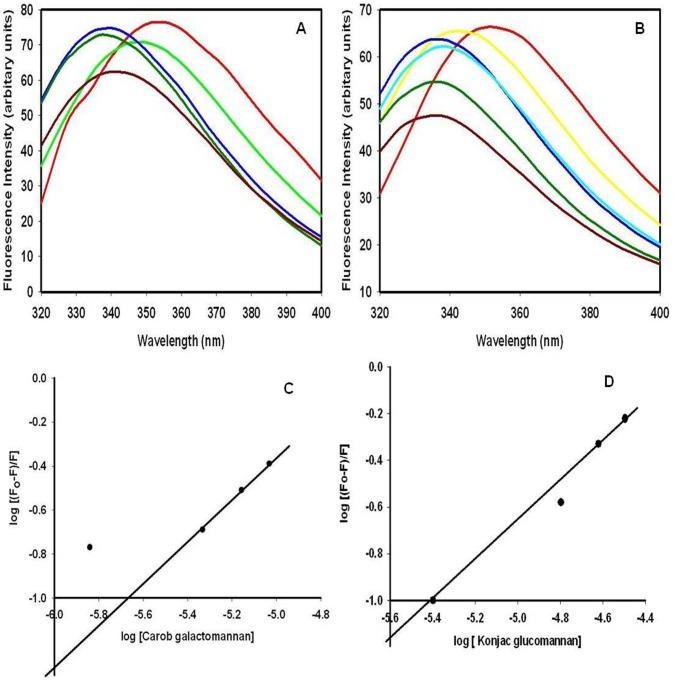
Tryptoptophan fluorescence emission spectrum of *Ct*CBM35 in presence of (A) carob galactomannan (%, w/v), represented in lines: (red) without polysaccharide, (in green) 0.01, (light blue) 0.04, (dark green) 0.06, (dark red) 0.08. (B) konjac glucomannan (red) without polysaccharide, (yellow) 0.01, (deep blue) 0.04, (light blue) 0.06, (dark green) 0.08, (dark red) 0.1. (C) Hill plot of log [(F_o_−F)/F] vs log [carob galactomannan] (D) Hill plot of 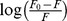 vs log [konjac glucomannan] used to derive association constant (*K*_a_).

Among the three aromatic amino acids tryptophan shows highest quantum yield and better stability facilitates its presence to utilize as a probe for fluorescence detection during polysaccharide binding with protein [17]. In presence of polysaccharides such as carob galactomannan and konjac glucomannan with their varying concentration from 0.01%–0.08% (w/v) significant blue shifts were observed. Binding of carob galactomannan and konjac glucomannan with *Ct*CBM35 displayed 21 nm peak shifts towards shorter wavelength of tryptophan emission spectra from λ_max_ 350 nm to 329 nm (Figure 4A and Figure 4B). The association constant (*K*_a_) of *Ct*CBM35 with carob galactomannan and konjac glucomannan were derived from Hill plot (Figure 4C and Figure 4D). From Hill plot and relative fluorescence intensities the values of 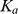 with carob galactomannan was found to be 11.4×10^4^ M^−1^ and with konjac glucomannan 14.3×10^4^ M^−1^ (Table 1). It was found that the 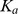 values approximately similar as derived earlier from affinity electrophoresis. Therefore, the fluorescence studies of polysaccharide binding of *Ct*CBM35 confirmed the results of affinity electrophoresis. The number of binding site concentrations (n) were derived from Stern Volmer equation and with carob galactomannan n = 0.79 and whereas with konjac glucomannan n = 0.80 (Table 1). It means both the polysaccharide has single binding site for *Ct*CBM35. Since *Ct*CBM35 displayed significant affinities for mannose derived polysaccharide in combination of galactose and glucose in their side and main chains, form the derived affinity constants the Gibb's free energy of binding were calculated using the equation:
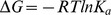

*where*, ΔG = Gibb's free energy, R = gas constant (Joule K^−1^ mole^−1^), T = Temperature in Kelvin, 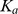 = association constant (M^−1^). The free energy binding of *Ct*CBM35 with carob galactomannan was −22.0 kJ mole^−1^ and with konjac glucomannan −25.0 kJ mole^−1^ (Table 1). The higher binding affinity and free energy of binding suggested that likely due to the simple molecular architecture of konjac glucomannan made an easy platform for *Ct*CBM35 than carob galactomannan, although both the polysaccharides have similar number of binding sites.

### Study of size of *Ct*CBM35 on binding with polysaccharide and Ca^2+^ by DLS

**Figure 5:**
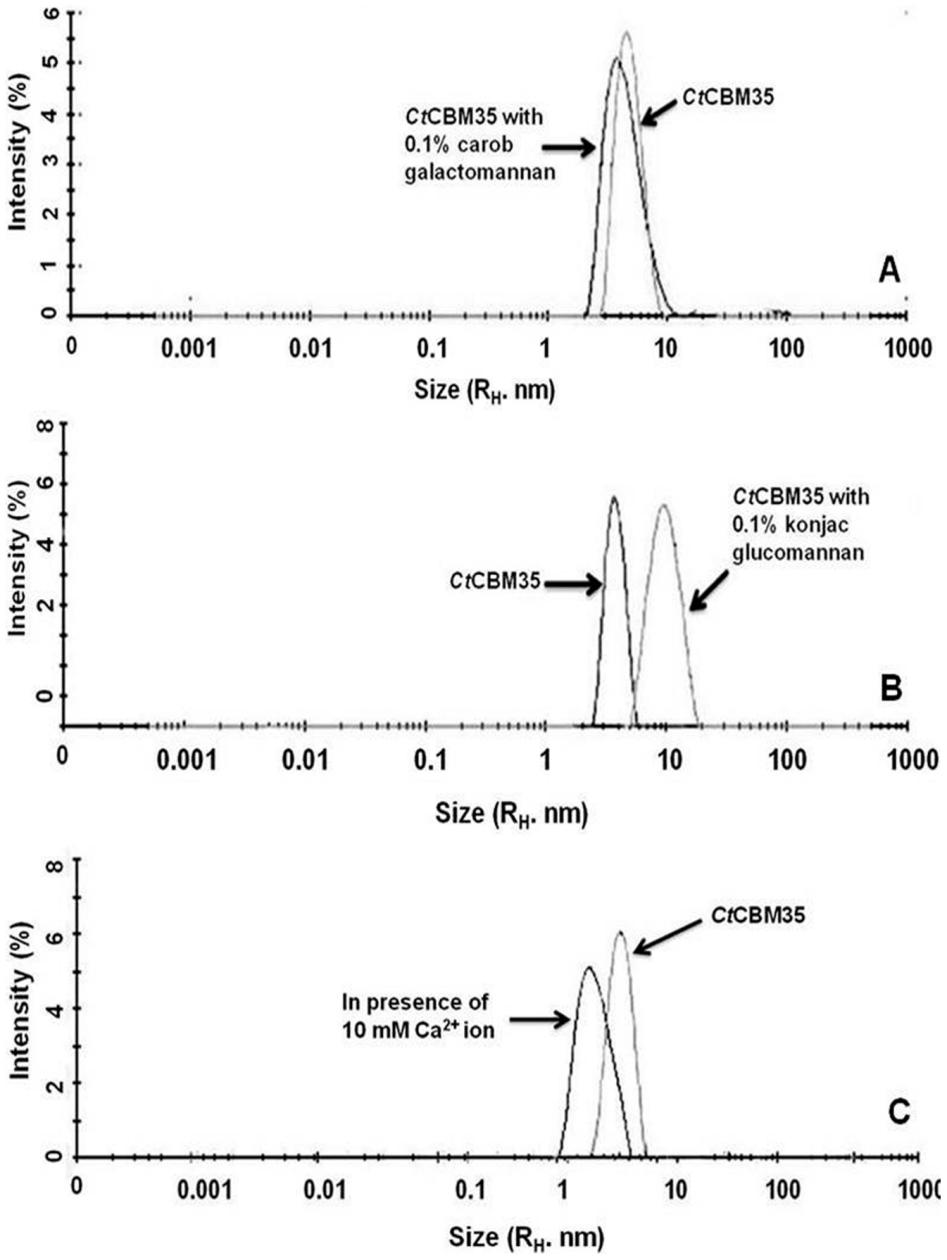
Dynamic light scattering of *Ct*CBM35 in conjugation with 0.1% (w/v) (A) carob galactomannan, (B) konjac glucomannan and (C) 10 mM Ca^2+^ ion.

Polysaccharides binding greatly influence the alterations in the dynamic environment of a protein. As measured from dynamic light scattering (DLS) the hydrodynamic radius (R_H_) of *Ct*CBM35 was found to be 4.25 nm which was in the acceptable range as this value is higher than theoretical R_H_, 2 nm for a 15 kDa protein. The R_H_ augmentation of *Ct*CBM35 was seen in presence of 0.1% (w v^−1^) carob galactomannan from 4.25 nm to 6 nm (Figure 5 A). In contrast, konjac glucomannan binding (0.1%, w v^−1^) exhibited much broader R_H_ of 8 nm (Figure 5 B). The augmentation of size was due to strong binding with konjac glucomannan leading to a stiffer structure and low random diffusion of the particles of protein and polysaccharides in the dynamic environment. Random diffusion of the particles measured in terms of random diffusion coefficient and is inversely proportional to R_H_. In presence of 10 mM Ca^2+^ ion, the hydrodynamic radius of *Ct*CBM35 was changed remarkably from 4.25 nm in absence of Ca^2+^ ion to 4.52 nm (Figure 5 C). In this case Ca^2+^ ion might bind with the amino acid residues of *Ct*CBM35 and imparted a stiffer orientation than the usual. Therefore, random diffusion of the system reduced and as a result the dynamic radius of *Ct*CBM35-Ca^2+^ complex was increased.

### Protein melting and molecular dynamics study of *Ct*CBM35 in presence of Ca^2+^ ion

**Figure 6:**
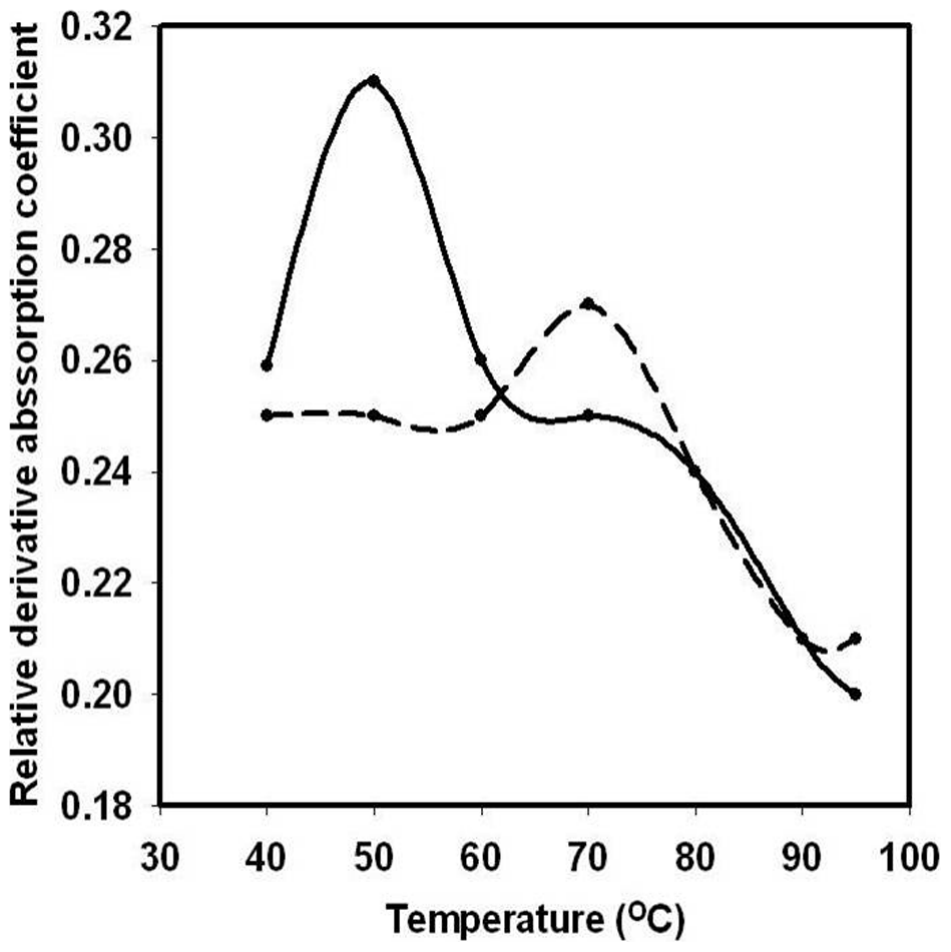
Protein melting curve of *Ct*CBM35 (—) in absence of 10 mM Ca^2+^ ion, (— —) in presence of 10 mM Ca^2+^ ion.

**Figure 7:**
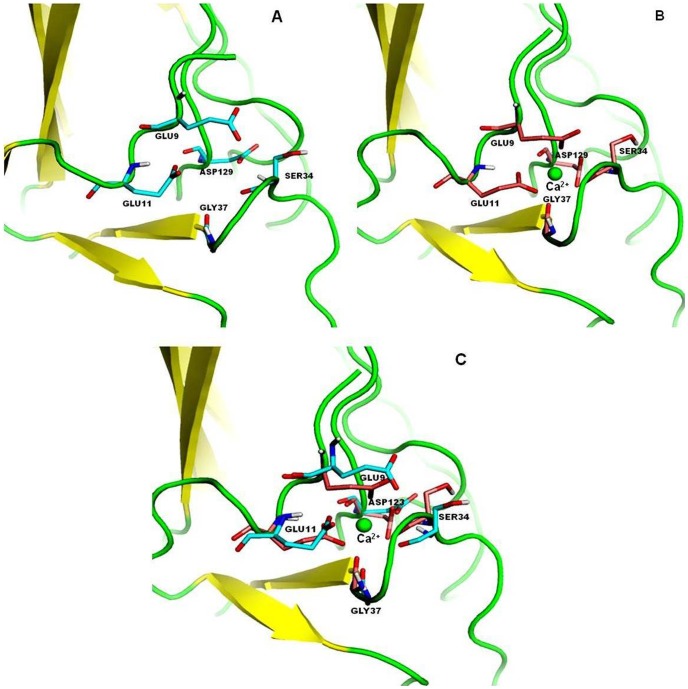
Amino acid residues of *Ct*CBM35 in the modeled structure (A) without Ca^2+^ ion (B) with Ca^2+^ ion (C) superimposed structure of both (A) and (B) showing the Ca^2+^ ion binding pocket to compare the altered positions of the amino acid residues in absence and presence of Ca^2+^ ion.

Melting of *Ct*CBM35 was studied in presence of additive Ca^2+^ ion to investigate the protein stability which perhaps, play a major role in polysaccharide recognition at higher temperature and improve the catalytic properties of catalytic modules. The melting of *Ct*CBM35 at λ_max_ 280 nm showed an unfolded peak at 50°C (Figure 6). In the presence of 10 mM CaCl_2_ as additive, the melting point of *Ct*CBM35 was shifted towards higher temperature at 70°C and the low melting peak at 50°C disappeared completely (Figure 6). Thus, Ca^2+^ ion played a key role in providing better stability of *Ct*CBM35 at higher melting temperature as compared to lack of additive. This analysis was further analyzed using PyMOL tool from the generated model of *Ct*CBM35. There was one Ca^2+^ ion binding pocket interacting with 7 amino acid residues *viz.* Glu 9, Glu 11, Ser 34, Gly 37, Asp 129. It was observed that negatively charged residues were predominant except Ser and Gly which interacted strongly with positively charged Ca^2+^ ions. These amino acid residues made coordinate bonds with Ca^2+^ ion and their orientation was changed remarkably. The comparative study of two *Ct*CBM35 models with unbound and bound Ca^2+^ ion (Figure 7 A and 7 B) displayed orientation of amino acid residues within the binding pocket. When both the models were superimposed (Figure 7 C), it was observed that the residues changed their orientations at their similar positions with Ca^2+^ ion bound state than the unbound structure. The bound state residues have less root mean square deviation (RMSD) value of 1.08 Å as compared to unbound state of 1.8 Å. This reduced RMSD value due to the stiffer binding with Ca^2+^ ion prevented less free movement at their bound state [29].

## Discussion

The cloned family 35 Carbohydrate binding module (*Ct*CBM35) from *Clostridium thermocellum* preferred binding with manno-configured polysaccharides. *Ct*CBM35 discriminated during carbohydrate selection showing its affinity only with manno-configured ligands among the manno and xylo-configured polysaccharides. Instead of β-1,4-mannose chain affinity the variance of ligand selection within the manno-configured polysaccharides was often observed. *Ct*CBM35 has higher affinity with konjac glucomannan than carob galactomannan. Rationale behind this selective affinity was due to the galactose unit in carob galactomannan likely interferes with the *Ct*CBM35 binding. Carob galactomannan is composed of a 1,4-β-linked D-mannan backbone to which single D-galactosyl units are attached to C-6 of D-mannosyl residues. Whereas glucomannan is a linear polysaccharide comprising 1,4-β-linked D-glucosyl and D-mannosyl residues. Therefore, *Ct*CBM35 was more glucomannan specific than galactomannan. This finding gained a new insight into the CBM35 family when compared with other mannan specific CBM35 from *Clostridium thermocellum* and *Cellvibrio japonicas* [30], [31] which were exo and endo acting to D-mannan chain of galactomannan only. However, the binding of *Ct*CBM35 with insoluble polysaccharides was quite insignificant. Low affinity against insoluble mannan was due to the inability of trans (as a discrete fold) form of *Ct*CBM35 to disrupt the inter chain interactions in mannan. This in contrast to some CBMs has the ability to disintegrate the surface of crystalline insoluble polysaccharides that potentiates higher binding to some soluble fractions of insoluble polysaccharides [32], [33]. Polysaccharide specificity by CBM35 family is probably due to the conserved hydrophobic aromatic residues that play a major role in polysaccharide binding [30]. Tryptophan being one of such residues has indole ring with intrinsic fluorescence property with higher quantum yield, displays fluorescence emission at 320 nm to 400 nm [17]. Polysaccharide binding changes the microenvironment of tryptophan due to conformational changes in protein. Usually in CBMs, the aromatic residues responsible for polysaccharide binding are lying in the hydrophobic core. Due to polysaccharide binding and direct interaction with tryptophan, the fluorescence emission is gradually decreased. The higher affinity of *Ct*CBM35 for konjac glucomannan masks the available tryptophan for fluorescence emission as compared to carob galactomannan. Thus, gradual fall in peak intensities were coupled with peak shifts (∼21 nm) due to altered conformation of native *Ct*CBM35. In dynamic light scattering, the larger particle size of *Ct*CBM35 is due to the polysaccharide binding. The cationic interaction of aromatic residues with carob galactomannan and konjac glucomannan insists *Ct*CBM35 domain alteration to more compact form reducing the random diffusion of the particles between polysaccharide and amino acid residues. Due to simpler structure of konjac glucomannan, the interaction with aromatic residues in the binding pocket of *Ct*CBM35 uphold strong binding as compared to carob galactomannan with substituted galactose side chain. The structure of *Ct*CBM35 gains its particle size in the conjunction with polysaccharide and the Brownian motion of light measured between the larger particles and ends up in a larger hydrodynamic area. Melting of a protein module is a cooperative process [34]–[36]. CBM acts as an independent subunit from the catalytic module at higher temperature. But divalent cation like Ca^2+^ ion makes polar contact with the amino acid residues away from the polysaccharide binding sites. This imparts stiffer orientation of the residues dragging more towards the ion and holding into a proper position of the residues even at varying physical parameters such as higher temperature, pH change etc with reduced RMSD value. Ca^2+^ ion attacks on the negatively charged amino acid side chains and do not allow their mobility at harsh environmental conditions due to which the protein folding remain intact [29]. Thus a recombinant thermostable glucomannan and galactomannan specific CBM35 from *Clostridium thermocellum* may be useful to enhance the activity by appended to a mannanase for higher degree of hydrolysis of complex manno-configured polysaccharides into simple sugars. Moreover, these findings might lead to comprehend both glucomannan and galactomanan specific CBM35 from *Clostridium thermocellum* which may play a potential role in biofuel production in conjunction with mannanase from mannan rich polysaccharides in future.
